# Design, Synthesis, and Biological Characterization of Macromolecular Ester Prodrugs of a Selective Cyclooxygenase‐2 Inhibitor

**DOI:** 10.1002/cmdc.202500691

**Published:** 2025-11-18

**Authors:** Mario Saletti, Marco Paolino, Jacopo Venditti, Germano Giuliani, Antonietta Rossi, Danilo D’Avino, Sara Perna, Ina Varfaj, Roccaldo Sardella, Antonio Macchiarulo, Samuele Maramai, Stefania Lamponi, Andrea Cappelli, Maurizio Anzini

**Affiliations:** ^1^ Dipartimento di Biotecnologie Chimica e Farmacia Università degli Studi di Siena Via Aldo Moro 2 53100 Siena Italy; ^2^ Dipartimento di Farmacia Scuola di Medicina e Chirurgia Università di Napoli “Federico II” Via D. Montesano 49 I‐80131 Napoli Italy; ^3^ Dipartimento di Scienze Farmaceutiche Università di Perugia Via Fabretti 48 06123 Perugia Italy

**Keywords:** anti‐inflammatory agents, COX‐2 inhibitors, ferulic acid, hyaluronic acid, prodrugs

## Abstract

Owing to the importance of tracing new routes in the development of macromolecular prodrugs, in the present work, two potential macromolecular ester prodrugs (i.e., **12a** and **13a**) of selective cyclooxygenase‐2 (COX‐2) inhibitor **7b** are designed and synthesized. In the design, two different oligo(ethylene glycol)‐based spacers are linked through a ferulate residue to the backbone hyaluronic acid (**HA**) showing a medium molar mass value (i.e., Mw = 270 kDa). The spacers are designed to differ in the sensitivity to the hydrolytic conditions so that the chemical hydrolysis of ferulate ester bond in **12a** is assumed to produce the corresponding ferulic acid derivative **12b**. On the other hand, the same reaction in **13a** leading to ferulate derivative **13b** could be accompanied by the hydrolysis of the second ester bond with the release of the selective COX‐2 inhibitor **7b**. The COX inhibitory activity of the newly synthesized compounds is evaluated in vitro, and macromolecular ester prodrugs **12a** and **13a** are found to be completely inactive together with hydrolysis product **12b**. Conversely, these in vitro studies reveal the intriguing COX‐2 inhibitory activity and selectivity of ferulate derivative **13b** related to macromolecular ester prodrug **13a**. Therefore, to obtain information on the hydrolysis process in different environments, hydrolysis studies are performed on macromolecular ester prodrug **13a** by using ^1^H NMR and UHPLC‐MS techniques. These studies show that severe hydrolytic conditions (i.e., aqueous NaOH solutions) promote the rapid release of potent and selective COX‐2 inhibitor **7b**, whereas in ammonium acetate buffer the release is slower. Overall, these results lead to envision possible applications of the design approach to the development of macromolecular ester prodrugs of all the drug molecules bearing hydroxyl groups in their structures.

## Graphical abstract

Two macromolecular ester prodrugs of a COX‐2 inhibitor are designed and synthesized. They are based on two different oligo(ethylene glycol) spacers linking the COX‐2 inhibitor to hyaluronic acid. The spacers are designed to differ in the sensitivity to the hydrolytic conditions. Severe hydrolytic conditions promote the rapid release of the COX‐2 inhibitor, whereas the release of the COX‐2 inhibitor in ammonium acetate buffer is slower.

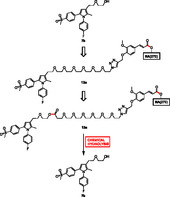

## 1 Introduction

Nonsteroidal anti‐inflammatory drugs (NSAIDs) represent one of the most used classes of pharmaceuticals for treating pathological conditions associated with inflammation, either by prescription or self‐medication. NSAIDs resulted particularly effective also in the management of acute and chronic pain and they are often referred to as “pain killers”.^[^ ^1^ ^]^ Accordingly, these drugs have found wide applications in the treatment of rheumatoid arthritis (RA) and osteoarthritis (OA),^[^ ^2^ ^]^ lupus erythematosus,^[^ ^3^ ^]^ fibromyalgia,^[^ ^4^ ^]^ intestinal bowel disease (IBD),^[^ ^5^ ^]^ dysmenorrhea,^[^ ^6^ ^]^ and inflammations in the urogenital tract^[^ ^7^ ^]^ and respiratory system.^[^ ^8^ ^]^ Moreover, postoperative or end‐stage cancer pain has been managed with combinations of NSAIDs and narcotic drugs.^[^ ^9^ ^,^ ^10^ ^]^

NSAIDs owe their analgesic and anti‐inflammatory actions to the inhibition of cyclooxygenases (COX), the enzymes responsible for the conversion of arachidonic acid (AA) into prostaglandins and other lipid mediators. These enzymes exist as two distinct isozymes: COX‐1, constitutively expressed in most tissues, and COX‐2, which is principally expressed in response to pro‐inflammatory stimuli such as cytokines.^[^ ^11^ ^]^ However, recent findings demonstrated that in specific districts of the human body, such as the central nervous system (CNS), the primary response is COX‐2 mediated while COX‐1 is involved in the secondary response, occurring for the perpetration of the inflammatory stimulus.^[^ ^12^, ^13^ ^–^ ^14^ ^]^

First generation NSAIDs (i.e., salicylates and propionic acids) generally inhibit both COX isoforms, thus hampering the synthesis of prostanoids mediated by COX‐1 and interfering with the functions of the gastric mucosa and renal homeostasis. Consequently, the prolonged use of NSAIDs is accompanied by severe gastrointestinal (GI) complications (mucosal damage, bleeding, etc.) and renal damage, representing a major drawback for this class of drugs.^[^ ^15^ ^]^ This has prompted the development of second generation NSAIDs, also known as coxibs, which are characterized by the selective inhibition of COX‐2 and by a significantly lower gastric toxicity.^[^ ^16^ ^]^ The connection between the selectivity on COX isoforms and reduced gastric side‐effects has also been reported for a few selective inhibitors of COX‐1.^[^ ^17^ ^]^

Aiming at novel anti‐inflammatory agents potentially useful in the therapeutic approach of OA,^[^ ^18^, ^19^ ^–^ ^20^ ^]^ our research group has recently reported hybrid molecules capable of both inhibiting COX‐2 and releasing nitric oxide (NO‐donors), also indicated as CINOD.^[^ ^20^ ^]^ These compounds were specifically designed to prevent cardiovascular and renal side effects commonly associated with the use of vicinal diaryl‐substituted heterocycles (VDHs), to which drugs such as rofecoxib and valdecoxib structurally belong.^[^ ^21^ ^]^

**Figure 1:**
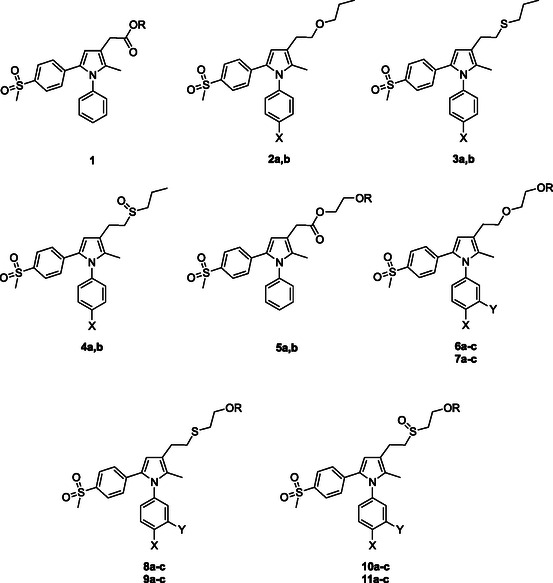
Development of 3‐substitued‐1,5‐diarylpyrroles: COX‐2 inhibitors (COXIB) **1–4**; dual COX‐2 inhibiting NO‐donors (CINOD) **5a**, **6a‐c** and their respectively active metabolites **5b**, **7a‐c**; thio‐analogs (thio‐CINOD) nitrooxy‐ and hydroxy ethyl sulfides **8a‐c, 9a‐c** and their respectively oxidation products nitrooxy‐ and hydroxyethyl sulfoxides **10a‐c**, **11a‐c**. Substituents: **2a**, **3a**, **4a**, X = H; **2b**, **3b**, **4b**, X = F; **5a**, R = NO_2_; **5b**, R = H; **6a**, **8a**, **10a**, X = Y = H, R = NO_2_; **6b**, **8b**, **10b**, X = F, Y = H, R = NO_2_; **6c**, **8c**, **10c**, X = Y = F, R = NO_2_; **7a**, **9a**, **11a**, X = Y = R = H; **7b**, **9b**, **11b**, X = F, Y = R = H; **7c**, **9c**, **11c**, X = Y = F, R = H.

However, the structural motif of two phenyl rings on contiguous atoms of a five‐ or six‐membered heterocyclic system makes VDHs versatile scaffolds for building compounds with peculiar clinical applications.^[^ ^22^ ^]^ In light of this, we performed an accurate investigation into the design and development of 3‐substituted‐1,5‐diarylpyrrole derivatives (**Figure** **1**).

These analogues include carboxylic acids and esters with general structure **1**,^[^ ^23^ ^]^ alkoxy ethers **2a,b**,^[^ ^24^ ^]^ alkyl sulfides **3a,b**, and sulfoxides **4a,b** ^[^ ^25^ ^]^ along with nitrooxyalkyl esters **5a,b,** ^[^ ^26^ ^]^ ethers **6a‐c**, and hydroxyethyl derivatives **7a‐c,** ^[^ ^27^ ^]^ all endowed with interesting biological features. Compared to the standard selective COX‐2 inhibitors (e.g., celecoxib), the novel compounds demonstrated similar or improved COX‐2 inhibitory activities.^[^ ^23^, ^24^, ^25^, ^26^ ^–^ ^27^ ^]^ The nitrooxy analogues **5a** and **6a‐c** acted as efficient NO‐donors, thus displaying significant vasorelaxant properties^[^ ^28^ ^]^ and confirming the effectiveness of the design. The encouraging results stimulated the synthesis of nitrooxy‐ and hydroxy ethyl sulfides **8a‐c** and **9a‐c**, along with their oxidation products **10a‐c** and **11a‐c** (namely thio‐CINOD).^[^ ^29^ ^]^

In our laboratories, we also carry out intensive studies for the development of polymers with a broad range of biological properties. In this context, special attention has been dedicated to hyaluronic acid (**HA**), a nonsulfated, naturally occurring *glycosaminoglycan* derived from the condensation of glucuronic acid with *N*‐acetylglucosamine.^[^ ^30^ ^]^ **HA** is found in all connective tissues and body fluids and represents the fundamental constituent of the extracellular matrix as well as a key component in articular cartilage and synovial fluid.^[^ ^31^, ^32^, ^33^ ^–^ ^34^ ^]^ Among its physiological roles, **HA** promotes tissue repair after injury and helps to dampen excessive loads on joints.^[^ ^35^ ^]^ It also affects tissue hydration, in correlation with its ability to retain large amounts of water, therefore representing an important lubricant supplement in eye and osteoarticular diseases.^[^ ^36^ ^]^ Accordingly, **HA** found interesting applications in drug delivery system (DDS) formulations^[^ ^37^ ^]^ and biocompatible hydrogels for medical and pharmaceutical purposes.^[^ ^37^, ^38^, ^39^, ^40^, ^41^, ^42^, ^43^, ^44^, ^45^, ^46^ ^–^ ^47^ ^]^

Recently, we developed hyaluronan derivatives by grafting **HA** with ferulic acid (**FA**) moieties bearing propargyl groups, thus obtaining **HA**‐**FA‐Pg** graft copolymers.^[^ ^48^ ^,^ ^49^ ^]^ These latter were used in the setup of a technology platform potentially useful for coating applications, as reported for the covalent coating of a polybenzofulvene cylindrical brush.^[^ ^50^, ^51^ ^–^ ^52^ ^]^ The optimized procedure was then applied to the surface coating of different nanostructures, including small unilamellar vesicles (SUVs),^[^ ^53^ ^]^ self‐assembling micelles,^[^ ^54^ ^]^ magnetic nanoparticles,^[^ ^55^ ^]^ and poly(propylene imine) (PPI) dendrimers.^[^ ^56^ ^]^ Lastly, **HA‐FA‐Pg** graft copolymers were combined with cross‐linking agents as a strategy for increasing structural complexity.^[^ ^57^, ^58^ ^–^ ^59^ ^]^

**Figure 2:**
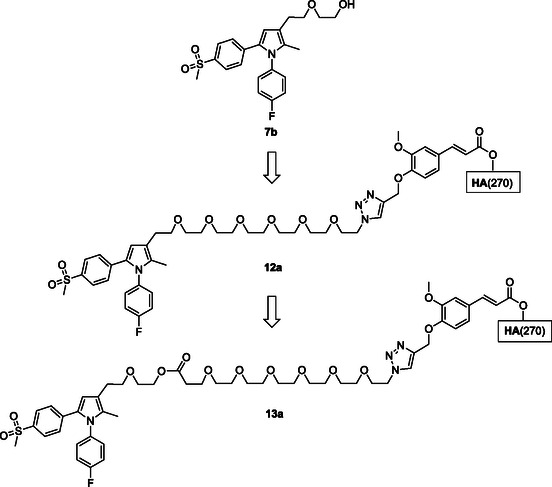
Development of PEGylated ester prodrugs of selective COX‐2 inhibitor **7b**.

Based on our well‐consolidated background, in the present work we investigate the development of macromolecular ester prodrugs of potent COX‐2 inhibitor **7b** as covalent conjugates of **HA** showing a medium molar mass value (i.e., Mw = 270 kDa) (**Figure** **2**).

Macromolecular prodrugs demonstrated to have powerful applications in biomedicine, since the nature and the molecular weight of the polymeric matrix can influence toxicity, solubility, pharmacokinetics, and body distribution of drugs for different treatments.^[^ ^60^ ^]^ This is particularly interesting for NSAIDs, since their conjugation and release form complex matrices could help overcome some of the limits connected to their systemic use, including gastrointestinal side‐effects.^[^ ^61^ ^]^ In the presence of the suitable trigger, NSAIDs will be released at variable rates to exert their anti‐inflammatory activity.

In the current approach, two PEGylated macromolecular ester prodrugs of selective COX‐2 inhibitor **7b** were designed to show two different oligo(ethylene glycol)‐based spacers covalently bound to **HA**(270) backbone through a ferulate residue. The key difference in the designed oligo(ethylene glycol)‐based spacers was the sensitivity to the hydrolytic conditions. Thus, the chemical hydrolysis of ferulate ester bond in **12a** was assumed to produce the corresponding FA derivative, whereas the same reaction in **13a** could be accompanied by the hydrolysis of the second ester bond with the release of the selective COX‐2 inhibitor **7b**.

## 2 Results and Discussion

### 2.1 Synthesis

**Scheme 1:**
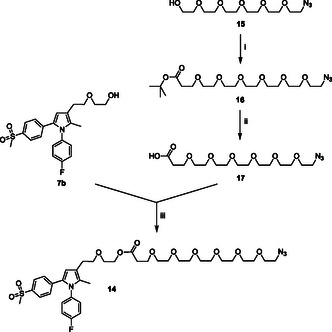
Preparation of clickable synthon **14**. Reagents: (i) Na^0^, *tert*‐butyl acrylate, dry THF; (ii) TFA; (iii), DCC, DMAP, dry THF.

The synthetic work started with the preparation of hydroxyethyl ether **7b** according to previously reported high‐yielding procedures.^[^ ^27^ ^]^ Then, a convergent synthetic strategy (**Scheme** **1**) was developed, involving the functionalization of **7b** with amphiphilic OEG‐based side chain **12** via an easily cleavable ester bond, to obtain clickable synthon **14**.

The mono‐azido derivative **15** was obtained according to previously reported synthetic procedures.^[^ ^58^ ^]^ Subsequently, the hydroxyl group of compound **15** was reacted with *tert*‐butyl acrylate in the presence of sodium in dry THF to yield derivative **16** bearing a *tert*‐butyl ester group, which was subjected to cleavage with trifluoroacetic acid (TFA), providing intermediate **17**. Afterward, an esterification reaction was performed, by means of the previous activation of the carboxylic functional group embedded in compound **17** with *N*,*N*′‐dicyclohexylcarbodiimide (DCC), in the presence of 4‐(dimethylamino)pyridine (DMAP) as the base, to give derivative **14** in a moderate yield (49%).

**Scheme 2:**
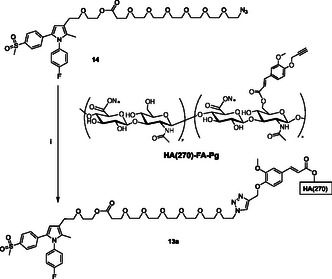
Click‐chemistry reaction of clickable synthon **14** with **HA(270)**‐**FA**‐**Pg** graft copolymer exhibiting ca. 20% grafting degree. Reagents: (i) CuSO_4_, sodium ascorbate, *tert*‐butanol, H_2_O.

**Scheme 3:**
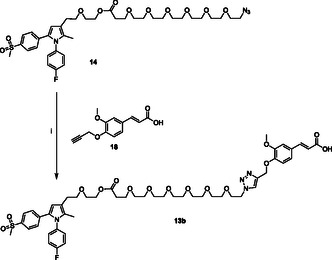
Click‐chemistry reaction of clickable synthon **14** with ferulate **18** bearing a clickable propargyl group. Reagents: (i) CuSO_4_, sodium ascorbate, *tert*‐butanol, H_2_O.

The clickable azido group of the resulting agent **14** was then used in the CuAAC reactions with **HA(270)**‐**FA**‐**Pg** graft copolymer showing a 20% grafting degree to obtain material **13a** (**Scheme** **2**) or with ferulate **18** bearing a clickable propargyl group^[^ ^58^ ^]^ to obtain acid **13b** (**Scheme** **3**). The catalytic species copper(I) was generated in situ from CuSO_4_ using sodium ascorbate as the reducing agent, to perform the CuAAC reaction under very mild conditions.

**Scheme 4:**
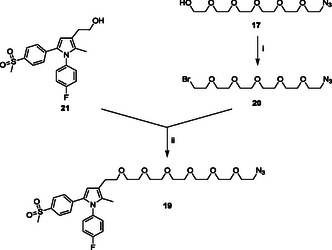
Preparation of clickable agent **19**. Reagents: (i) PBr_3_, dry CH_2_Cl_2_; (ii) NaH, dry THF.

Similarly, a convergent synthetic strategy (**Scheme** **4**) was exploited to obtain clickable agent **19**.

Briefly, the hydroxyl group of the previously synthesized mono‐azido derivative **17** ^[^ ^58^ ^]^ was replaced with a bromine atom by using PBr_3_ in dry dichloromethane to afford compound **20**. Once obtained, reactive intermediate **20** was employed in the alkylation of the hydroxylic moiety of derivative **21** ^[^ ^26^ ^,^ ^62^ ^]^ in the presence of sodium hydride as the base in dry THF to obtain clickable synthon **19**.

**Scheme 5:**
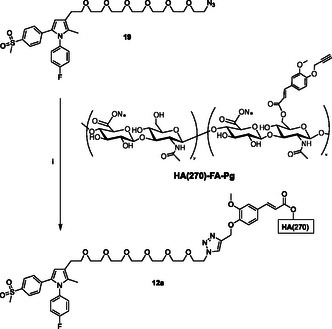
Click‐chemistry reaction of clickable synthon **19** with **HA(270)**‐**FA**‐**Pg** graft copolymer exhibiting 20% grafting degree. Reagents: (i) CuSO_4_, sodium ascorbate, *tert*‐butanol, H_2_O.

**Scheme 6:**
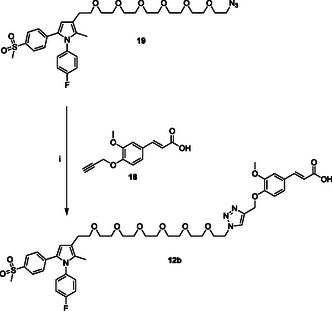
Click‐chemistry reaction of clickable synthon **19** with ferulate **18** bearing a clickable propargyl group. Reagents: (i) CuSO_4_, sodium ascorbate, *tert*‐butanol, H_2_O.

The clickable azido group of the resulting agent **19** was then used in the CuAAC reaction with **HA(270)**‐**FA**‐**Pg** graft copolymer showing ca. 20% grafting degree to obtain **12a** (**Scheme** **5**), or with ferulate **18** bearing a clickable propargyl group^[^ ^58^ ^]^ to obtain acid **12b** (**Scheme** **6**).

Compounds **12b** was designed and synthesized as the most probable product of the hydrolysis of the corresponding **HA**‐based material **12a**.

### 2.2 In Vitro COX‐2 Inhibitory Activity and Selectivity of the Newly Synthesized Compounds

The in vitro COX‐1 and COX‐2 inhibitory activity of **12a**,**b**, **13a**,**b**, **14**, and **19** was measured by evaluating the inhibition of PGE_2_ production in cell‐based assays employing murine monocyte/macrophage J774 cell lines in comparison with reference compounds **7b**, **21**, and celecoxib, and the results are summarized in **Table** **1**.

**Table 1:** Comparison of the in vitro COX‐1 and COX‐2 inhibitory activity (J774 murine macrophage assay) of the newly synthesized compounds 12a,b, 13a,b, 14, and 19 with the one shown by reference selective COX‐2 inhibitors 7b, 21, and celecoxib.

Compound	COX‐1 IC_50_ [μM]a)	COX‐2 IC_50_ [μM]a)	COX‐1/COX‐2 [SI]b)
**12a**	>10	>10	–
**12b**	>10	>10	–
**13a**	>10	>10	–
**13b**	>10	0.05	>200
**14**	>10	0.015	>666.7
**19**	>10	0.5	>20
**7b** c)	>10	0.004	>2500
**21** d)	>10	0.07	>142.8
**Celecoxib** e)	3.84	0.061	>63.0

a) Results are expressed as the mean of three experiments of the % inhibition of PGE_2_ production by the test compounds with respect to control samples;

b) In vitro COX‐2 selectivity index [IC_50_(COX‐1)/IC_50_(COX‐2)];

c) See ref. [27];

d) See ref. [24];

e) See ref. [26].

Selectivity toward COX‐2, referred to as the Selectivity Index (SI), is expressed as the ratio of the concentrations needed to inhibit the activity of both isoenzymes by 50% (IC_50_), that is, IC_50_(COX‐1)/IC_50_(COX‐2). All tested compounds didn’t inhibit the production of PGE2 via COX‐1 in AA‐stimulated J774 macrophages.

The most important result was that the designed macromolecular ester prodrugs **12a** and **13a** were found to be completely inactive in inhibiting the PGE_2_ production via COX‐2 in LPS‐stimulated macrophages. This required that hydrolysis was mandatory to obtain the possible release of bioactive compounds from the macromolecular ester prodrugs. On the other hand, the biological features of the corresponding hydrolysis products **12b** and **13b** were very different since **12b** was completely devoid of COX‐2 inhibitory activity, while **13b** showed a very interesting IC_50_ value in the nanomolar range (i.e., 50 nM). Interestingly, the same trend was observed when clickable agents **14** and **19** were considered, with ester derivative **14** (IC_50_ = 15 nM) being more potent than ether derivative **19** (IC_50_ = **500** nM). However, the presence of an easily hydrolyzable ester bond in compounds **14** and **13b** could play a role in permitting the release of the highly potent COX‐2 inhibitor **7b** during the in vitro studies. If this could be true for small molecule esters **13b** and **14**, it did not occur in macromolecular ester prodrug **13a**. All these considerations led us to consider the importance of the possible hydrolysis process, which could lead to release the bioactive compounds from the macromolecular ester prodrug **13a**.

### 2.3 Hydrolysis Studies

**Figure 3:**
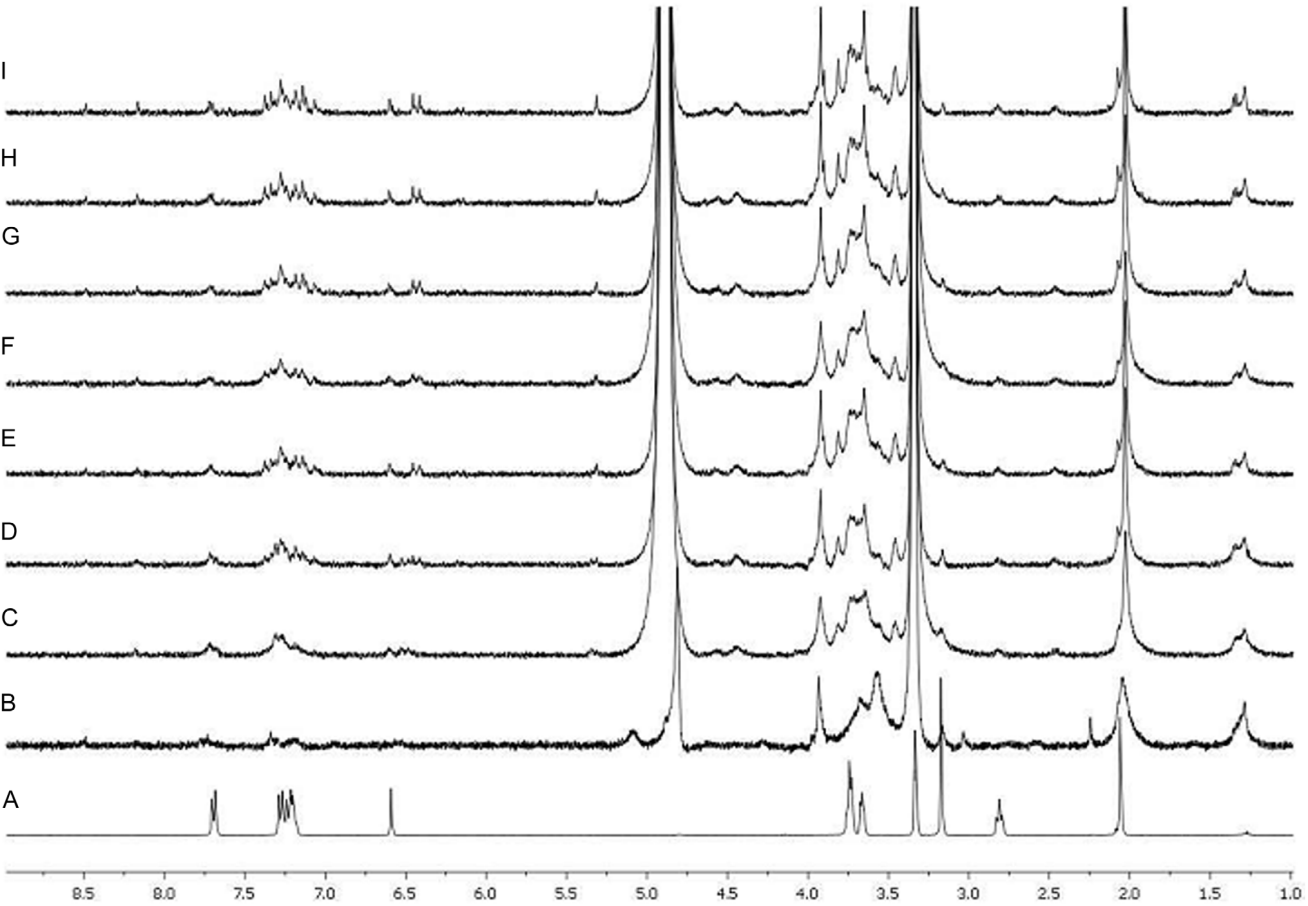
Hydrolysis studies of **13a**. ^1^H NMR spectra of A) compound **7b** (0.140 mg) in deuterated water‐deuterated methanol (50:50 v/v, 1.0 mL); B) a dispersion of **13a** (1.0 mg) in deuterated water‐deuterated methanol (50:50 v/v, 1.0 mL) before the treatment and C) immediately after (*t* = 0 min) the treatment with NaOD 10% at room temperature into an NMR tube; D) after 20 min at room temperature; E) after 40 min at room temperature; F) after 60 min at room temperature; G) after 80 min at room temperature; H) after 100 min at room temperature, and I) after 120 min at room temperature.

Hydrolysis studies from **13a** were performed in the first instance employing ^1^H NMR. In detail, a dispersion of **13a** (1.0 mg) in a mixture of deuterated water‐deuterated methanol (50:50 v/v, 1.0 mL) was treated with NaOD 10% at room temperature into an NMR tube and ^1^H NMR spectra were recorded at regular time intervals. The comparison of the spectra (**Figure** **3**) showed that the broad signals of macromolecular ester prodrug **13a** (Figure 3B), after the NaOD addition, progressively became sharper so that it was possible to appreciate the appearance of signals that could be considered diagnostic of the presence of compound **7b** (compare Figure 3I with Figure 3A).

In order to obtain accurate information on the hydrolysis process involving macromolecular ester prodrug **13a**, hydrolysis studies were also performed through ultra‐high performance liquid chromatographic analysis coupled to mass spectrometric detection (UHPLC‐MS).

Compounds were solubilized in a water (either plain buffered or alkaline, according to the specific set of analyses)/methanol (50:50, v/v) solution. A water/methanol (50:50, v/v) solution (blank sample) was injected before each set of analyses to evaluate both the incidental presence of carry‐over and the presence of system peaks.

The effects of the exposition of **13a** to hydrolytic conditions was investigated in two different systems: the first relying upon the use of a sodium hydroxide (NaOH) solution, the second implying the use of a buffer system consisting of ammonium acetate.

Exactly weighted **13a** (1.0 mg) was solubilized in either an aqueous NaOH solution (40% w/v) or an aqueous ammonium acetate (50 mM, pH 7.4) solution, to have the final concentration of 1 mg mL^–1^ (1000 ppm), adding the same volume (50:50, v/v) of methanol. In both cases, an aliquot of this solution (10 µL) was further diluted with the same solvent to obtain a final concentration of 20 ppm. The obtained solutions were then submitted to the UHPLC‐MS analysis and, through the above‐mentioned hydrolysis conditions, were analyzed immediately, and then (i) after 40 min and 120 min the NaOH containing solution, and (ii) every 20 min for six consecutive times the ammonium acetate containing solution.

**Figure 4:**
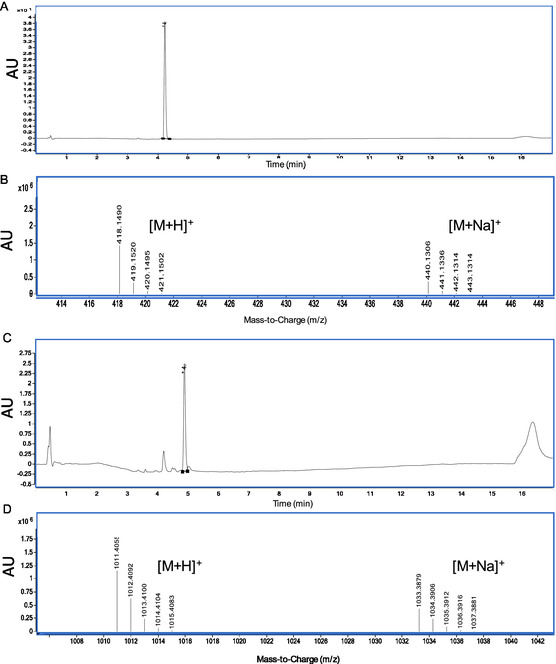
A) UV chromatograms at 350 nm obtained from the analysis of the pure compound of **7b** and C) **13b**. B) MS spectra obtained from the analysis of the standard compound **7b** and D) **13b**.

Peak identity in the hydrolysis products was established by comparing (i) the retention times obtained in the analysis of pure standards and (ii) MS spectra. The analysis results of pure standards are shown in **Figure** **4**, where the chromatograms of the pure standards, as well as the isotopic pattern distribution for the [M + H]^+^ and [M + Na]^+^ adducts, are reported.

**Figure 5:**
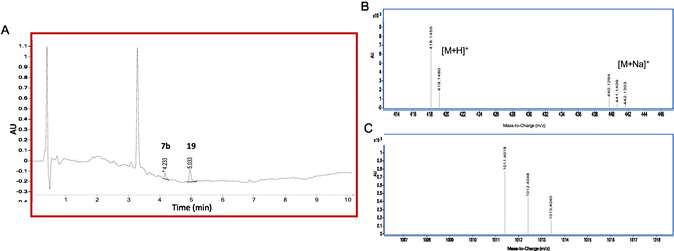
A) UV chromatogram at 350 nm obtained after the hydrolysis of **13a** material with ammonium acetate buffer. B) MS spectrum of the peak at 4.232 min and C) at 5.003 min, respectively referred to compounds **7b** and **13b** according to the MS spectra of the standard compounds reported in Figure 4.

Regarding macromolecular ester prodrug **13a**, only the presence of **7b** was found as a result of the hydrolysis with NaOH, while compound **13b** was completely absent (or not detectable). Instead, the hydrolysis with ammonium acetate led to the constant presence of derivative **7b** as the main product, with subtle amounts of **13b** detected only immediately after the hydrolysis protocol (at t_0_). Also in this case, as evident from the UV chromatogram depicted in **Figure** **5**A and the MS spectra in Figure 5B,C, a strict adherence in terms of both retention times and isotopic pattern distributions with the standard compounds occurred.

Hydrolysis with NaOH produced higher releases of compound **7b** in comparison to those obtained with the use of ammonium acetate. Moreover, in the presence of the buffer, a slight increase of the hydrolysis extent was found to occur over the time.

## 3 Conclusions

Two macromolecular ester prodrugs (i.e., **12a** and **13a**) of selective COX‐2 inhibitor **7b** were designed and synthesized by taking into consideration two different oligo(ethylene glycol)‐based spacers linked through a ferulate residue to the backbone **HA** showing a medium molar mass value (i.e., Mw = 270 kDa). In the design of these novel prodrugs, we focused our attention on the sensitivity to the hydrolytic conditions as the key difference in the designed oligo(ethylene glycol)‐based spacers. In fact, the chemical hydrolysis of ferulate ester bond in **12a** was assumed to produce the corresponding FA derivative **12b**, whereas the same reaction in **13a** (leading to ferulate derivative **13b**) could be complemented by the hydrolysis of the second ester bond with the release of the selective COX‐2 inhibitor **7b**. The evaluation of the in vitro COX inhibitory activity of the newly synthesized compounds revealed the lack of activity in macromolecular ester prodrugs **12a** and **13a** and also of the ferulate derivative **12b**, the probable hydrolysis product deriving from **12a**. On the other hand, these in vitro studies revealed the interesting COX‐2 activity and selectivity of ferulate derivative **13b** deriving from macromolecular ester prodrug **13a**. Thus, hydrolysis studies were performed on this latter employing ^1^H NMR and UHPLC‐MS techniques in order to obtain information on the hydrolysis process in different environments. The results of these studies revealed that severe hydrolytic conditions were capable of promoting the rapid release of potent and selective COX‐2 inhibitor **7b**, whereas in ammonium acetate buffer the release was slower. Overall, these results led to envision possible applications of the design approach to the development of macromolecular ester prodrugs of all the drug molecules bearing hydroxyl groups in their structures.

## 4 Experimental Section

4.1

4.1.1

##### Materials and Methods

All reagents and solvents were obtained from Sigma–Aldrich (Taufkirchen, Germany) and were used without further purification. Sodium salt of hyaluronic acid, with an average molecular weight (Mw) of 260 ± 10 kDa and a polydispersity index (Mw/Mn) of 3.1 ± 0.1 [**HA**(270)], was purchased from Biophil Italia SpA (Origgio, Italy) and used without further purifications.^[^ ^48^ ^]^ NaOH and ammonium acetate used for the hydrolysis studies were purchased from Merck Life Science (Merck KGaA, Darmstadt, Germany). Merck TLC aluminum sheets and silica gel 60 F_254_ were employed for TLC. NMR spectra were recorded with a Bruker DRX‐400 AVANCE spectrometer in the indicated solvents (residual peak of the solvent as the internal standard). The chemical shift (*δ*) values are reported in ppm and those of the H–H coupling constants (*J*) in Hz. Splitting patterns are described as singlet (s), doublet (d), triplet (t), multiplet (m), and broad (br). An Agilent 1100 LC/MSD running with an electrospray source (ESI) was used in mass spectrometry measurements.

##### Materials and Methods: Preparation of HA(270)‐FA‐Pg Graft Copolymers

**HA(270)‐FA‐Pg** graft copolymers were synthesized by following the previously described procedure,^[^ ^57^ ^]^ starting from **HA** showing a medium molar mass value (i.e., Mw = 270 kDa).

##### Materials and Methods: Preparation of Compounds 7b, 15, 18, and 21

Compounds **7b**, **15, 18**, and **21** were synthesized according to previously described procedures, and their spectroscopic and analytical data were consistent with those we have already reported.^[^ ^26^ ^,^ ^27^ ^,^ ^58^ ^,^ ^62^ ^,^ ^63^ ^]^

##### Materials and Methods: tert‐Butyl 1‐azido‐3,6,9,12,15,18‐hexaoxahenicosan‐21‐oate (16)

To a solution of mono‐azido derivative **15** ^[^ ^58^ ^]^ (1.03 g, 3.34 mmol) in dry THF (40 mL), Na^0^ (8.0 mg, 0.35 mmol) and *tert*‐butyl acrylate (4.89 mL, 33.4 mmol) were added in the sequence. The reaction mixture, after being stirred for 1 h at room temperature under a nitrogen atmosphere, was concentrated under reduced pressure. Then, the oily residue was purified by flash chromatography with ethyl acetate‐petroleum ether (8:2) as the eluent to give the expected compound **16** (1.05 g, yield 72%) as a pale‐yellow oil. ^1^H NMR (400 MHz, CDCl_3_): 1.41 (s, 9H), 2.46 (t, *J* = 6.6, 2H), 3.33–3.37 (m, 2H), 3.58–3.69 (m, 24H). MS (ESI): *m/z* 458.0 [M + Na^+^].

##### Materials and Methods: 1‐Azido‐3,6,9,12,15,18‐hexaoxahenicosan‐21‐oic acid (17)

A mixture of compound **16** (704 mg, 1.62 mmol) in TFA (1.7 mL, 22.6 mmol) was stirred for 30 min at room temperature under a nitrogen atmosphere. The reaction mixture was then added to toluene and concentrated under reduced pressure. Flash chromatography purification of the resulting oily residue with ethyl acetate‐methanol (9:1) as the eluent gave the expected compound **17** (217 mg, yield 35%) as a yellow oil. ^1^H NMR (400 MHz, CDCl_3_): 2.61 (br s, 2H), 3.39 (t, *J* = 4.9, 2H), 3.60–3.81 (m, 24H). MS (ESI): *m/z* 378.9 [M−H^+^].

##### Materials and Methods: 2‐(2‐(1‐(4‐Fluorophenyl)‐2‐methyl‐5‐(4‐(methylsulfonyl)phenyl)‐1H‐pyrrol‐3‐yl)ethoxy)ethyl 1‐azido‐3,6,9,12,15,18‐hexaoxahenicosan‐21‐oate (14)

To a solution of derivative **17** (190 mg, 0.50 mmol) in dry THF (20 mL), DCC (103 mg, 0.50 mmol), DMAP (15.3 mg, 0.125 mmol), and compound **7b** ^[^ ^27^ ^]^ (209 mg, 0.50 mmol) were added in the sequence. The reaction mixture, after being stirred overnight at room temperature under a nitrogen atmosphere, was concentrated under reduced pressure, and the organic residue was dissolved in ethyl acetate and treated with a saturated solution of NaCl. The organic layer was then dried over anhydrous sodium sulfate, filtered, and the solvent evaporated in vacuo. The crude product was purified by flash chromatography with ethyl acetate‐methanol (9:1) as the eluent to give the expected compound **14** (191 mg, yield 49%) as a yellow oil. ^1^H NMR (400 MHz, CDCl_3_): 2.04 (s, 3H), 2.61 (t, *J* = 6.5, 2H), 2.77 (t, *J* = 7.3, 2H), 3.00 (s, 3H), 3.36–3.40 (m, 2H), 3.58–3.76 (m, 28H), 4.26 (m, 2H), 6.43 (s, 1H), 7.10–7.17 (m, 6H), 7.65–7.68 (m, 2H). MS (ESI): *m/z* 801.3 [M + Na^+^].

##### Materials and Methods: 1‐Azido‐17‐bromo‐3,6,9,12,15‐pentaoxaheptadecane (20)

To a solution of compound **17** ^[^ ^58^ ^]^ (506 mg, 1.65 mmol) in dry CH_2_Cl_2_ (20 mL), PBr_3_ (0.19 mL, 1.98 mmol) was added dropwise. After being stirred at room temperature under a nitrogen atmosphere for 2 h, the reaction mixture was concentrated under reduced pressure, and the organic residue was partitioned between dichloromethane and water. The organic layer was then dried over anhydrous sodium sulfate, filtered, and concentrated under reduced pressure. The resulting oily residue was purified by flash chromatography using ethyl acetate‐petroleum ether (9:1) as the eluent to give the expected compound **20** (158 mg, yield 26%) as a yellow oil. ^1^H NMR (400 MHz, CDCl_3_): 3.36–3.40 (m, 2H), 3.46 (t, *J* = 6.3, 2H), 3.64–3.67 (m, 18H), 3.80 (t, *J* = 6.3, 2H). MS (ESI): *m/z* 392.0*,* 394.0 [M + Na^+^].

##### Materials and Methods: 3‐(20‐Azido‐3,6,9,12,15,18‐hexaoxaicosyl)‐1‐(4‐fluorophenyl)‐2‐methyl‐5‐(4‐(methylsulfonyl)phenyl)‐1H‐pyrrole (19)

To a solution of compound **21** ^[^ ^27^ ^]^ (232 mg, 0.62 mmol) in dry THF (6.0 mL), NaH (89.5 mg, 3.73 mmol) and **20** (230 mg, 0.62 mmol) were added in the sequence. The reaction mixture, after being stirred for 5 h at reflux temperature under a nitrogen atmosphere, was cooled at 0 °C, concentrated under reduced pressure. The resulting residue was dissolved in ethyl acetate and treated with water. The organic layer was then dried over anhydrous sodium sulfate, filtered, and the solvent evaporated in vacuo. The crude product was purified by flash chromatography with ethyl acetate as the eluent to give the compound **19** (77 mg, yield 19%) as an orange oil. ^1^H NMR (400 MHz, CD_3_OD): 2.08 (s, 3H), 2.79 (t, *J* = 7.0, 2H), 3.08 (s, 3H), 3.38 (m, 2H), 3.63–3.70 (m, 24H), 6.56 (s, 1H), 7.22–7.29 (m, 6H), 7.71 (d, *J* = 8.5, 2H). MS (ESI): *m/z* 685.3 [M + Na^+^].

##### Materials and Methods: (E)‐3‐(4‐((1‐(27‐(1‐(4‐Fluorophenyl)‐2‐methyl‐5‐(4‐(methylsulfonyl)phenyl)‐1H‐pyrrol‐3‐yl)‐21‐oxo‐3,6,9,12,15,18,22,25‐octaoxaheptacosyl)‐1H‐1,2,3‐triazol‐4‐yl)methoxy)‐3‐methoxyphenyl)acrylic acid (13b)

Under an inert atmosphere, a 10 mL flask was charged with *tert*‐butanol (2.0 mL), water (2.0 mL), and a solution of CuSO_4_ pentahydrate (12.5 mg, 0.050 mmol) in 0.50 mL of water. A 1 m solution of sodium ascorbate (100 mg, 0.050 mmol) in water (0.50 mL) was then added and 2.5 mL of the resulting mixture was used as the catalyst. A mixture of the clickable intermediate **14** (55 mg, 0.071 mmol) and compound **18** ^[^ ^19^ ^]^ (18 mg, 0.078 mmol) in *tert*‐butanol (2.0 mL) and water (2.0 mL) was treated with the catalyst solution (2.5 mL) and the reaction mixture was stirred at room temperature for 6 h and then concentrated under reduced pressure. The organic residue was dissolved in ethyl acetate and treated with a saturated solution of NH_4_Cl. The organic layer was then dried over anhydrous sodium sulfate, filtered, and the solvent was concentrated under reduced pressure. The resulting residue was purified by flash chromatography using ethyl acetate‐methanol (8:2 v/v) as the eluent to give the expected compound **13b** (46 mg, yield 64%) as a yellow oil. ^1^H NMR (400 MHz, CDCl_3_): 2.03 (s, 3H), 2.61 (t, *J* = 6.5, 2H), 2.76 (t, *J* = 7.3, 2H), 3.00 (s, 3H), 3.47–3.63 (m, 20H), 3.65–3.74 (m, 6H), 3.84 (t, *J* = 5.0, 2H), 3.88 (s, 3H), 4.26 (t, *J* = 4.8, 2H), 4.52 (t, *J* = 4.8, 2H), 5.33 (s, 2H), 6.32 (br d, 1H), 6.43 (s, 1H), 7.04–7.15 (m, 9H), 7.56–7.72 (m, 3H), 7.80 (s, 1H). MS (ESI): *m/z* 1033.3 [M + Na^+^].

##### Materials and Methods: (E)‐3‐(4‐((1‐(20‐(1‐(4‐Fluorophenyl)‐2‐methyl‐5‐(4‐(methylsulfonyl)phenyl)‐1H‐pyrrol‐3‐yl)‐3,6,9,12,15,18‐hexaoxaicosyl)‐1H‐1,2,3‐triazol‐4‐yl)methoxy)‐3‐methoxyphenyl)acrylic acid (12b)

Under an inert atmosphere, a 10 mL flask was charged with *tert*‐butanol (2.0 mL), water (2.0 mL), and a solution of CuSO_4_ pentahydrate (12.5 mg, 0.050 mmol) in 0.50 mL of water. A 1 M solution of sodium ascorbate (100 mg, 0.050 mmol) in water (0.50 mL) was then added and 2.5 mL of the resulting mixture was used as the catalyst. A mixture of the intermediate **19** (54 mg, 0.0815 mmol) and compound **18** ^[^ ^19^ ^]^ (21 mg, 0.090 mmol) in *tert*‐butanol (2.0 mL) and water (2.0 mL) was treated with the catalyst solution (2.5 mL) and the reaction mixture was stirred at room temperature for 6 h and then concentrated under reduced pressure. The organic residue was dissolved in ethyl acetate and treated with a saturated solution of NH_4_Cl. The organic layer was then dried over anhydrous sodium sulfate, filtered, and the solvent was concentrated under reduced pressure. The resulting residue was purified by flash chromatography using ethyl acetate‐methanol (8:2 v/v) as the eluent to give the expected compound **12b** (27 mg, yield 37%) as an orange oil. ^1^H NMR (400 MHz, CDCl_3_): 2.03 (s, 3H), 2.77 (t, *J* = 7.4, 2H), 2.99 (s, 3H), 3.49–3.68 (m, 22H), 3.82–3.85 (m, 2H), 3.88 (s, 3H), 4.49–4.53 (m, 2H), 5.33 (s, 2H), 6.31 (d, *J* = 15.8, 1H), 6.43 (s, 1H), 7.10 (m, 9H), 7.65 (m, 3H), 7.80 (s, 1H). MS (ESI): *m/z* 917.3 [M + Na^+^].

##### Materials and Methods: General Procedure of Click‐Chemistry Reaction of HA(270)‐FA‐Pg Graft Copolymer for the Synthesis of 13a and 12a

Under an inert atmosphere, a 10 mL flask was charged with *tert*‐butanol (2.0 mL), water (2.0 mL), and a solution of CuSO_4_ pentahydrate (12.5 mg, 0.050 mmol) in 0.50 mL of water. A 1 M solution of sodium ascorbate (100 mg, 0.050 mmol) in water (0.50 mL) was then added and 1.0 mL of the resulting mixture was used as the catalyst. A mixture of the medium molecular weight **HA(270)**‐**FA**‐**Pg** graft copolymer showing a grafting degree of ca. 20–25% (250 mg) and the appropriate clickable agents **14** or **19** (see the amounts below)^[^ ^57^ ^]^ in *tert*‐butanol (25 mL) and water (25 mL) was treated with the catalyst solution (1.0 mL) and the reaction mixture was stirred overnight at room temperature and then concentrated under reduced pressure. Purification of the residue by washing in sequence with ethanol (50 mL) and diethyl ether (3 × 10 mL) gave the corresponding materials, which were dried under reduced pressure.

##### Materials and Methods: Macromolecular Ester Prodrug 13a

The title macromolecular ester prodrug was prepared by the general procedure cited above starting from **HA(270)‐FA‐Pg‐20** (250 mg)^[^ ^57^ ^]^ and clickable agent **14** (107.5 mg, 0.138 mmol) to obtain **13a** (214 mg) as a white solid. ^1^H NMR (400 MHz, D_2_O): Figure 5.

##### Materials and Methods: Macromolecular Ester Prodrug 12a

The title macromolecular ester prodrug was prepared by the general procedure cited above starting from **HA(270)‐FA‐Pg‐20** (250 mg)^[^ ^57^ ^]^ and clickable agent **19** (91.5 mg, 0.138 mmol) to obtain **12a** (330 mg) as an off‐white solid.

##### Hydrolysis Studies

The analysis was carried out with the UHPLC‐MS system Agilent 1290 Infinity II combined with the Agilent 6560 mass spectrometer (Agilent Technologies Inc., Santa Clara, California, USA). The chromatographic separation was performed using a ZORBAX RRHD Eclipse Plus C18 column (50 × 2.1 mm, 1.8 µm, 95 Å, Agilent Technologies Inc.). UHPLC eluent A was water (LC‐MS grade, LiChrosolv, Supelco) with 0.1% (v/v) formic acid (LC‐MS grade, LiChropur, Supelco) while eluent B was methanol (LC‐MS grade, LiChrosolv, Supelco). The optimized gradient program was the following: 0–3 min, 5% (v/v) B; 3–13 min, 5–70% (v/v) B; 13–15 min, 95% (v/v) B; 15–16 min, 95% (v/v) B; 11–16 min, 5% (v/v) B, 16–18 min 5% B (columnequilibration/conditioning). The column temperature was set at 25 °C and the flow rate at 0.3 mL min^−1^. The injection volume was 5 µL. The UV‐Vis chromatograms were recorded at 250, 280, 320, and 350 nm. For MS detection, the Dual AJS ESI source operated in positive ion mode. The gas temperature was set at 300 °C with a flow of 5 L min^–1^, while the sheath gas temperature was set at 350 °C with a flow of 11 L min^–1^. The nebulizer pressure was set at 35 psi and the capillary and fragmentor voltages were 3500 V and 400 V, respectively. Ions were acquired in the 50–1500 m z^–1^ range.

##### In Vitro Inhibition of COX‐1 and COX‐2 Enzymes

The in vitro COX‐1 and COX‐2 inhibition of **7b**, **12a,b**, **13a,b**, **14**, **19**, **21** was evaluated through cell‐based assay employing murine monocyte/ macrophage J774 cell lines. The cell line was grown in DMEM supplemented with 2 mM glutamine, 25 mM HEPES, 100 units mL^–1^ penicillin, 100 μg mL^–1^ streptomycin, 10% fetal bovine serum (FBS), and 1.2% sodium pyruvate. Cells were plated in 24‐well culture plates at a density of 2.5 × 10^5^ cells mL^–1^ and allowed to adhere at 37 °C in 5% CO_2_ for 2 h. Immediately before the experiments, the culture medium was replaced with fresh medium, and cells were stimulated as previously described.^[^ ^64^ ^]^ The evaluation of COX‐1 inhibitory activity was achieved by pretreating cells with test compounds (10 μM) for 15 min and then incubating them at 37 °C for 30 min with 15 μM arachidonic acid to activate the constitutive COX. At the end of the incubation, the supernatants were collected to measure PGE_2_ levels by an enzyme immuno‐assay (EIA). To evaluate COX‐2 activity, cells were stimulated for 24 h with Escherichia coli lipopolysaccharide (LPS, 10 μg mL^–1^) to induce COX‐2, in the absence or presence of test compounds (0.001–10 μM). The supernatants were collected for the measurement of PGE_2_ by means of EIA. Celecoxib was utilized as a reference compound for the selectivity index. Triplicate wells were used for the various treatment conditions in the cell culture assay throughout the experiments. Results are expressed as the mean of three experiments of the % inhibition of PGE_2_ production by test compounds with respect to control samples. Data fit was obtained using the sigmoidal dose–response equation (variable slope) (GraphPad software). The IC_50_ values were calculated by the GraphPad Instat program (GraphPad software).

## Conflict of Interest

The authors declare no conflict of interest.

## Author Contributions

The manuscript was written through the contributions of all authors. All authors have given approval to the final version of the manuscript.

## Data Availability

The data that support the findings of this study are available from the corresponding author upon reasonable request.

## References

[1] T. Grosser , E. M. Smyth , G. A. A. FitzGerald , (Eds: L. Brunton , B. A. Chabner , B. C. Knollmann ), 13th edition, Zanichelli Editore 2019, 727–753.

[2] J.‐Y. Reginster , O. Bruyère , P. G. Conaghan , T. McAlindon , C. Cooper , Drugs & Aging. 2019, 36, 3. 31073919 10.1007/s40266-019-00659-8 PMC6509090

[3] M. Ostensen , P. M. Villiger , Lupus 2000, 9, 566. 11035430 10.1191/096120300678828794

[4] S. Derry , P. J. Wiffen , W. Häuser , M. Mücke , T. R. Tölle , R. F. Bell , R. A. Moore , Cochrane Database Syst. Rev. 2017, 3, CD012332. 28349517 10.1002/14651858.CD012332.pub2 PMC6464559

[5] X.‐P. Miao , J.‐S. Li , Q. Ouyang , R.‐W. Hu , Y. Zhang , H.‐Y. Li , Cochrane Database Syst. Rev. 2014, 10, CD007744. 10.1002/14651858.CD007744.pub2 PMC11200115 25340915

[6] S. L. Corson , R. J. Bolognese , J. Reprod. Med. 1978, 20, 246. 353274

[7] A. Kahokehr , R. Vather , A. Nixon , A. G. Hill , BJU Int. 2013, 111, 304. 23356748 10.1111/j.1464-410X.2012.11559.x

[8] C. Warren‐Gash , J. A. Udell , J. Infect. Dis. 2017, 215, 497. 28158625 10.1093/infdis/jiw604

[9] M. N. A. Khan , Y. S. Lee , Med. Res. Rev. 2011, 31, 161. 19967720 10.1002/med.20182

[10] J. Rømsing , S. Møiniche , Acta Anaesthesiol. Scand. 2004, 48, 525. 15101847 10.1111/j.0001-5172.2004.00379.x

[11] J. R. Vane , R. M. Botting , Am. J. Med. 1998, 104, 2S. 10.1016/s0002-9343(97)00203-9 9572314

[12] R. Calvello , D. D. Lofrumento , M. G. Perrone , A. Cianciulli , R. Salvatore , P. Vitale , F. De Nuccio , L. Giannotti , G. Nicolardi , M. A. Panaro , A. H. Scilimati , Front. Neurol. 2017, 8, 251. 28649222 10.3389/fneur.2017.00251 PMC5465243

[13] R. Calvello , M. A. Panaro , M. L. Carbone , A. Cianciulli , M. G. Perrone , P. Vitale , P. Malerba , A. Scilimati , Pharmacol. Res. 2012, 65, 137. 22001217 10.1016/j.phrs.2011.09.009

[14] P. Vitale , A. Panella , A. Scilimati , M. G. Perrone , Med. Res. Rev. 2016, 36, 641. 27111555 10.1002/med.21389

[15] G. Kauffman , Gastroenterology 1989, 96, 606. 2491826 10.1016/s0016-5085(89)80056-3

[16] D. L. DeWitt , Mol. Pharmacol. 1999, 55, 625. 10101019

[17] M. G. Perrone , D. D. Lofrumento , P. Vitale , F. De Nuccio , V. La Pesa , A. Panella , R. Calvello , A. Cianciulli , M. A. Panaro , A. P. Scilimati , Pharmacology, 2015, 95, 22. 25591798 10.1159/000369826

[18] A. Giordani , M. Biava , M. Anzini , V. Calderone , L. C. Rovati , Diaryl‐2‐alkylpyrrole‐3‐Substituted Nitro Esters, Selective COX‐2 Inhibitors and Nitric Oxide Donors, 2012, 1, WO2012032479A1.

[19] A. Fioravanti , L. Tinti , N. A. Pascarelli , A. Di Capua , A. Lamboglia , A. Cappelli , A. Biava , A. Giordani , S. Niccolini , M. Galeazzi , M. Anzini , J. Pharmacol. Sci. 2012, 120, 6. 22878602 10.1254/jphs.12016fp

[20] P. N. Geusens , Expert Opin. Biol. Ther. 2009, 9, 649. 19392579 10.1517/14712590902926071

[21] S. X. Sun , K. Y. Lee , C. T. Bertram , J. L. Goldstein , Curr. Med. Res. Opin. 2007, 23, 1859. 17605893 10.1185/030079907X210561

[22] M. A. Barmade , R. B. Ghuge , Vicinal Diaryl Heterocyclic System: A Privileged Scaffold in the Discovery of Potential Therapeutic Agents. Vicinal Diaryl Substituted Heterocycles, Elsevier, Amsterdam / New York 2018, 1–20.

[23] M. Biava , G. C. Porretta , A. Cappelli , S. Vomero , F. Manetti , M. Botta , L. Sautebin , A. Rossi , F. Makovec , M. Anzini , J. Med. Chem. 2005, 48, 3428. 15857149 10.1021/jm049121q

[24] M. Anzini , M. Rovini , A. Cappelli , S. Vomero , F. Manetti , M. Botta , L. Sautebin , A. Rossi , C. Pergola , C. Ghelardini , M. Norcini , A. Giordani , F. Makovec , P. Anzellotti , P. Patrignani , M. S. Biava , J. Med. Chem. 2008, 51, 4476. 18598017 10.1021/jm800084s

[25] A. Reale , S. Brogi , A. Chelini , M. Paolino , A. Di Capua , G. Giuliani , A. Cappelli , G. Giorgi , G. Chemi , A. Grillo , M. Valoti , L. Sautebin , A. Rossi , S. Pace , C. la Motta , L. di Cesare Mannelli , E. Lucarini , C. Ghelardini , M. S. Anzini , Bioorg. Med. Chem. 2019, 27, 115045. 31427145 10.1016/j.bmc.2019.115045

[26] M. Biava , G. C. Porretta , G. Poce , C. Battilocchio , S. Alfonso , M. Rovini , S. Valenti , G. Giorgi , V. Calderone , A. Martelli , L. Testai , L. Sautebin , A. Rossi , G. Papa , C. Ghelardini , L. di Cesare Mannelli , A. Giordani , P. Anzellotti , A. Bruno , P. Patrignani , M. Anzini , J. Med. Chem. 2011, 54, 7759. 21992176 10.1021/jm200715n

[27] M. Anzini , A. Di Capua , S. Valenti , S. Brogi , M. Rovini , G. Giuliani , A. Cappelli , S. Vomero , L. Chiasserini , A. Sega , G. Poce , G. Giorgi , V. Calderone , A. Martelli , L. Testai , L. Sautebin , A. Rossi , S. Pace , C. Ghelardini , L. di Cesare Mannelli , V. Benetti , A. Giordani , P. Anzellotti , M. Dovizio , P. Patrignani , M. Biava , J. Med. Chem. 2013, 56, 3191. 23534442 10.1021/jm301370e

[28] A. Martelli , L. Testai , M. Anzini , A. Cappelli , A. Di Capua , M. Biava , G. Poce , S. Consalvi , A. Giordani , G. Caselli , L. Rovati , C. Ghelardini , P. Patrignani , L. Sautebin , M. C. Breschi , V. Calderone , Pharmacol. Res. 2013, 78, 1. 24083950 10.1016/j.phrs.2013.09.008

[29] M. Saletti , S. Maramai , A. Reale , M. Paolino , S. Brogi , A. Di Capua , A. Cappelli , G. Giorgi , D. D’Avino , A. Rossi , C. Ghelardini , L. Di Cesare Mannelli , R. Sardella , A. Carotti , G. Woelkart , B. Klösch , C. Bigogno , G. Dondio , M. Anzini , Eur J. Med. Chem 2022, 241, 114615. 35932568 10.1016/j.ejmech.2022.114615

[30] K. T. Dicker , L. A. Gurski , S. Pradhan‐Bhatt , R. L. Witt , M. C. Farach‐Carson , X. H. Jia , Acta Biomater. 2014, 10, 1558. 24361428 10.1016/j.actbio.2013.12.019 PMC3960342

[31] D. H. Lee , J.‐H. Oh , J. H. Chung , J. Dermatol. Sci. 2016, 83, 174. 27378089 10.1016/j.jdermsci.2016.05.016

[32] P. Hildebrandt , Biomed. Tech. (Berl) 2002, 47 Suppl 1 Pt, 1, 476. 12451898 10.1515/bmte.2002.47.s1a.476

[33] G. D. Prestwich , D. M. Marecak , J. F. Marecek , K. P. Vercruysse , M. R. Ziebell , J. Cont. Release 1998, 53, 93. 10.1016/s0168-3659(97)00242-3 9741917

[34] G. D. Prestwich , J.‐W. Kuo , Curr. Pharm. Biotechnol. 2008, 9, 242. 18691083 10.2174/138920108785161523

[35] A. Fallacara , E. Baldini , S. Manfredini , S. Vertuani , Polymers 2018, 10, 701. 30960626 10.3390/polym10070701 PMC6403654

[36] M. Cohen , D. Joester , B. Geiger , L. Addadi , ChemBioChem 2004, 5, 1393. 15457530 10.1002/cbic.200400162

[37] S. Vasvani , P. Kulkarni , D. Rawtani , Int. J. Biol. Macromol. 2020, 151, 1012. 31715233 10.1016/j.ijbiomac.2019.11.066

[38] S. Trombino , C. Servidio , F. Curcio , R. Cassano , Pharmaceutics 2019, 11, 407. 31408954 10.3390/pharmaceutics11080407 PMC6722772

[39] A. Gilarska , J. Lewandowska‐Łańcucka , W. Horak , M. Nowakowska , Colloids Surf. B Biointerfaces 2018, 170, 152. 29902729 10.1016/j.colsurfb.2018.06.004

[40] S. Khunmanee , Y. Jeong , H. Park , J. Tissue Eng. 2017, 8, 2041731417726464. 28912946 10.1177/2041731417726464 PMC5590699

[41] X. Xu , A. K. Jha , D. A. Harrington , M. C. Farach‐Carson , X. Jia , Soft Matter 2012, 8, 3280. 22419946 10.1039/C2SM06463D PMC3299088

[42] Y. Xue , H. Chen , C. Xu , D. Yu , H. Xu , Y. Hu , RSC Adv. 2020, 10, 7206. 35493875 10.1039/c9ra09271d PMC9049836

[43] J. A. Burdick , G. D. Prestwich , Adv. Mater. 2011, 23, 41. 10.1002/adma.201003963 PMC3730855 21394792

[44] A. Borzacchiello , L. Russo , B. M. Malle , K. Schwach‐Abdellaoui , L. Ambrosio , Biomed. Res. Int. 2015, 2015, 871218. 26090451 10.1155/2015/871218 PMC4452290

[45] Y. Luo , K. R. Kirker , G. D. Prestwich , J. Control. Release 2000, 69, 169. 11018555 10.1016/s0168-3659(00)00300-x

[46] L. A. Pérez , R. Hernández , J. M. Alonso , R. Pérez‐González , V. Sáez‐Martínez , Biomedicines 2021, 9, 1113. 34572298 10.3390/biomedicines9091113 PMC8466770

[47] A. M. Mamaligka , K. Dodou , Gels 2024, 10, 54. 38247777 10.3390/gels10010054 PMC10815332

[48] M. Licciardi , C. Scialabba , G. Giammona , M. Paolino , A. Cappelli , J. Nanopart. Res. 2017, 19, 197.

[49] V. Razzano , M. Paolino , A. Reale , G. Giuliani , R. Artusi , G. Caselli , M. Visintin , F. Makovec , A. Donati , F. Villafiorita‐Monteleone , C. Botta , A. Cappelli , ACS Omega 2017, 2, 5453. 31457813 10.1021/acsomega.7b00789 PMC6644839

[50] A. Cappelli , M. Paolino , A. Reale , V. Razzano , G. Grisci , G. Giuliani , A. Donati , C. Bonechi , S. Lamponi , R. Mendichi , S. Battiato , F. Samperic , F. Makovecd , M. Licciardi , L. Depau , C. Botta , RSC Adv. 2018, 8, 5864. 35539623 10.1039/c7ra12543g PMC9078255

[51] M. Paolino , M. Licciardi , C. Savoca , G. Giammona , L. Modica De Mohac , A. Reale , G. Giuliani , H. Komber , A. Donati , G. Leone , A. Magnani , M. Anzini , A. Cappelli , Pharmaceutics 2019, 11, 675. 31842344 10.3390/pharmaceutics11120675 PMC6956235

[52] A. Atrei , C. Innocenti , S. Lamponi , S. Paesano , G. Leone , A. Reale , M. Paolino , A. Cappelli , Mater. Sci. Eng. C. 2020, 107, 110271. 10.1016/j.msec.2019.110271 31761218

[53] R. Mendichi , A. Giacometti Schieroni , Curr. Trends Polym. Sci. 2001, 6, 17.

[54] P. J. Wyatt , Anal. Chim. Acta 1993, 272, 1.

[55] W. Li , F. Xue , R. Cheng , Polymer (Guildf) 2005, 46, 12026.

[56] M. Paolino , P. Varvarà , M. Saletti , A. Reale , M. Gentile , E. Paccagnini , G. Giuliani , H. Komber , M. Licciardi , A. Cappelli , J. Appl. Polym. Sci. 2023, 140, e53300.

[57] M. Saletti , M. Paolino , L. Ballerini , G. Giuliani , G. Leone , S. Lamponi , M. Andreassi , C. Bonechi , A. Donati , D. Piovani , A. Giacometti Schieroni , A. Magnani , A. C.‐C. Cappelli , Pharmaceutics 2022, 14, 1041. 35631626 10.3390/pharmaceutics14051041 PMC9146110

[58] F. Terracina , M. Saletti , M. Paolino , J. Venditti , G. Giuliani , C. Bonechi , M. Licciardi , A. Cappelli , Gels 2024, 10, 91. 38391421 10.3390/gels10020091 PMC10887560

[59] M. Saletti , S. Pepi , M. Paolino , J. Venditti , G. Giuliani , C. Bonechi , G. Leone , A. Magnani , C. Rossi , A. Cappelli , Gels 2024, 10, 751. 39590107 10.3390/gels10110751 PMC11594237

[60] J. Alex , C. Weber , C. Guerrero‐Sanchez , U. S. Schubert , Prog. Polym. Sci. 2024, 155, 101855.

[61] G. Vadnerkar , S. Dhaneshwar , Curr Drug Discov Technol. 2013, 10, 16. 22725691

[62] A. Di Capua , C. Sticozzi , S. Brogi , M. Brindisi , A. Cappelli , L. Sautebin , A. Rossi , S. Pace , C. Ghelardini , L. Di Cesare Mannelli , G. Valacchi , G. Giorgi , A. Giordani , G. Poce , M. Biava , M. Anzini , Eur. J. Med. Chem. 2016, 15, 99. 10.1016/j.ejmech.2015.12.044 26774035

[63] A. Cappelli , M. Paolino , G. Grisci , V. Razzano , G. Giuliani , A. Donati , C. Bonechi , R. Mendichi , S. Battiato , F. Samperi , C. Scialabba , G. Giammona , F. Makovece , M. Licciardi , Polym. Chem. 2016, 7, 6529.

[64] A. Rossi , A. Ligresti , R. Longo , A. Russo , F. Borrelli , L. Sautebin , Phytomedicine 2002, 9, 530. 12403162 10.1078/09447110260573164

